# Analysis of the development trend of Chinese seafood imports from Southeast Asia after the Fukushima-Daiichi radioactive treated water discharge from Japan

**DOI:** 10.1371/journal.pone.0334879

**Published:** 2025-10-29

**Authors:** Xiaojun Qi, Liguo Gao

**Affiliations:** 1 Area studies, South China University of Technology, Guangzhou, Guangdong, China; 2 School of Literature and Communication, Zhaoqing University, Zhaoqing, Guangdong, China; 3 School of Foreign Languages, South China University of Technology, Guangzhou, Guangdong, China; Universiti Teknologi Malaysia, MALAYSIA

## Abstract

Japan’s decision to discharge the Fukushima-Daiichi radioactive treated water into the Pacific Ocean has drawn widespread international concern regarding radioactive contamination of seafood and its impact on the marine ecosystem and human health. China’s seafood import trade is confronted with a potentially significant threat. In the favorable circumstances created by China’s suspension of seafood imports from Japan, This article analyzes the impact of Japan’s radioactive treated water discharge on seafood safety and human health, as well as the importance of the Chinese market in the international trade of seafood, and analyzes the seafood export data from Southeast Asian countries to China from 2018 to 2024. A significant increase in seafood exports from countries such as Indonesia, Myanmar, and the Philippines to China was observed. Conversely, there has been a notable decline in the export volumes from Vietnam and Thailand. This paper proposes some proactive strategies for Southeast Asian seafood exports to China, including actively familiarizing with China’s import regulations and standards, optimizing export strategies, cultivating a positive and favorable product image, aligning with the needs of Chinese enterprises, and ensuring food safety.

## Introduction

The Fukushima-Daiichi nuclear power plant in Japan started discharging radioactive treated water into the sea [1]. Subsequently, Fukushima Nuclear Sewage Disposal Plan of Japan commenced, and it will last for nearly 30 years. After the Fukushima nuclear accident in Japan, the issue of the treated water discharge has attracted widespread global attention. This, in particular, has exerted a significant impact on the seafood import trade of neighboring nations, China being a notable and important example.

Contaminated wastewater contains various radioactive substances, and poses a grave threat to the marine ecosystem and human health. These radioactive substances have the potential to accumulate in aquatic organisms, thereby posing a substantial risk to human health if consumed. According to the latest report from the Japanese government, 621 radioactive isotopes were found in the existing nuclear water tanks in Fukushima [2]. The concentration of tritium- a radionuclide -reached about 860 TBq2, which is alarming level that far exceeds the prescribed standards [3].

Greenpeace highlighted the large amount of strontium-90 residues in the Fukushima nuclear sewage. This is one of the most hazardous and dangerous radionuclides. It is readily absorbed and accumulated in human bones, causing a higher risk of leukemia or blood cancer [3]. According to documents publicly provided by Tokyo Electric Power Company in Japan, in the 65,000 tons of water processed by the Advanced Liquid Processing System (ALPS) at the Fukushima-Daiichi Nuclear Power Station, the content of strontium-90 in the wastewater exceeded the regulatory standard by 100 times, and in some cases, it exceeded 20,000 times [2]. Carbon-14 cesium-137, iodine-129, and cobalt-60 and other radioactive materials are also present in the Fukushima wastewater, which pose significant environmental and health risks [3].

Therefore, the international community has expressed strong concerns and opposition to Japan’s decision. China, as a major importer of seafood, has similarly been impacted by this incident. In order to safeguard the health and safety of its citizens, China has implemented a comprehensive set of measures, which encompass the reinforcement of sampling inspections and laboratory tests, and the creation of a traceability system for imported goods. And the import of seafood from Japan was suspended.

The discharge of nuclear radioactive treated water has significant implications for the seafood import trade in neighboring countries, potentially leading to import restrictions and public health concerns. In particular, China, as one of the main importers of Japanese products, its seafood safety and trade volume have been significantly affected. China is the world’s largest seafood trading nation and the second largest importer, with its seafood imports ranking just behind the United States [4]. According to the latest research, China’s seafood imports have significantly contributed to the growth of the global aquatic product trade [4].

The growth rate of China’s aquatic product imports is also much higher than that of exports. As a traditional advantageous export product, China’s aquatic product trade has long been in surplus. In 2022, China’s seafood trade experienced a historic shift from a surplus to a deficit, with imports reaching $23.69 billion, marking the first time the country has seen a trade deficit in this sector. Japans seafood, including various types of seafood, occupies an important share of China’s seafood import trade [4]. According to statistics, over 97% of scallop imports into China come from Japan [4].

Consequent upon the discharge of contaminated wastewater from Japan, General Administration of Customs of the People’s Republic of China (GACC) declared suspension of the import of aquatic product, such as seafood, originating from Japan with effect from August 24, 2023 [5]. This decision has effectively curtailed the export of Japanese food potentially contaminated by radioactive substances to China, thereby safeguarding public health and life. The potential risk of contaminated wastewater discharge has a serious impact on China’s seafood import trade, and has profoundly impacted the volume of seafood imported from Southeast Asia into China. This article examines the impact of Japan’s discharge of the Fukushima-Daiichi radioactive treated water in August 2023 on the volume of seafood trade, using the data from September 2018 to August 2023 (S1 Table) and projections for September 2023 to August 2024 (S2 Table).

## Materials and methods

To evaluate the potential impact of the Fukushima Nuclear Sewage Disposal Plan on China’s seafood import trade, we employed a comprehensive analysis using both qualitative and quantitative analysis methods, incorporating data on import volumes, market responses.

The comprehensive review of existing literature and reports, related to the Fukushima Nuclear Sewage Disposal Plan and the hazards of radioactive substances to humans and marine life, is very important. Focusing on the types and concentrations of radioactive substances in the discharged wastewater, it could help us to understand the potential health risks associated with these substances.

Next we analyzed the trade data of China’s seafood imports, particularly focusing on imports from Japan and other Southeast Asian countries. And the data sourced from GACC is accurate and authoritative. We analyzed and compared the trends in import volumes before and after the announcement of the Fukushima Nuclear Sewage Disposal Plan.

Furthermore, we interviewed experts and industry insiders to gain insights into the potential long-term effects of the disposal plan on the seafood import trade of China and Southeast Asian countries. Their perspectives provided valuable insights into the potential challenges and opportunities confronting the industry. The recommendations formed on these basis perspectives are very reliable and worth practicing, regarding the export of seafood from Southeast Asian countries to China.

And the statistical software to analyze the collected data is very necessary, revealing trends and patterns within China’s seafood import trade. This analysis, drawing on recent trade data and expert insights, quantifies the potential impact of the Fukushima Nuclear Sewage Disposal Plan on seafood trade imports and exports of China and Southeast.

### Seafood import trade and threat from Japan’s discharge

#### Seafood and seafood trade.

Seafood, as defined by the Harmonized System Code (HS code), refer to the general term of aquatic animal and plant products and their processed products that are available for consumption or use in marine and freshwater fishery production, such as fish, shellfish, shrimp, chilled meat, seaweed and algae, etc. This paper classifies seafood through the international general HS code combined with the code regulations of GACC [6].

The seafood trade includes the import and export of various seafood. According to the UNCOMTRADE database coding system [7], the classification of seafood based on the HS code, combined with the regulations of the General Administration of Customs of the People’s Republic of China (GACC), allows for a more detailed understanding and management of seafood in trade.

These seafood HS codes are used to identify different types of seafood, covering various aquatic organisms and products, including fish, crustaceans and mollusks, etc., categorized into four groups with 262 items. The main category is, the second largest category: fish, crustaceans, mollusc, and other aquatic invertebrates, containing live, fresh, cold, frozen, dry, salted, salted, smoked seafood suitable for human consumption.

In the statistics of newspaper articles, trade data for aquatic products often refers to seafood under the second category of the HS coding system, while ignoring the processed seafood classified under the first, third, and fourth categories. These categories also account for a significant proportion. This paper compensates for such neglect, and the data collected includes not only the second category but also contains the first, third and fourth categories, which mainly involves seafood processing, such as seaweed and other related products suitable for human consumption; Seagrass and other algae that are not suitable for human consumption; Fish made or kept; sturgeon caviar and egg substitutes.

#### The important position of seafood in Chinese consumer goods.

The seafood trade holds a significant and growing position within China’s consumer goods sector. As one of the largest producers and consumers of seafood in the world, China has a vast demand for seafood, which has directly promoted the rapid development of the global aquaculture industry. Seafood is not only a crucial component of the daily diet of Chinese residents, but also holds a pivotal role in the national economy, forming a significant part of the dietary structure of Chinese residents. The quality and safety are directly related to national health and consumer confidence.

According to the data released by GACC in 2022, China’s cumulative import of seafood was 4.54 million tons, an increase of 20% year-on-year, with a cumulative import value of $22.1 billion, an increase of 40.6% compared to the previous year. China is one of the world’s largest importers of seafood, with its share in the global seafood trade increasing year by year.

In terms of international trade, China is an important participant in the global trade of seafood. Through imports and exports, China is closely intertwined with the aquaculture industry of various countries worldwide, forming a vast international network for the trade of seafood. This not only provides Chinese consumers with more diversified options of seafood but also brings new opportunities and challenges to the development of China’s domestic aquaculture industry.

China occupies a pivotal position in the international seafood trade. Through its import and export activities, China is closely intertwined with the aquaculture industries of various countries worldwide, establishing an extensive international network for the trade of seafood. This network not only expands Chinese consumers a broader range of aquatic product options but also presents new opportunities and challenges for the growth of China’s domestic aquaculture industry.

The importance of China’s seafood import trade is not only reflected in its supply guarantee for the domestic market but also lies in its impact on the global Marine ecological security and the international trade pattern.

After the Fukushima nuclear accident in 2011, China temporarily halted the imports of seafood from Japan, resulting in a significant decline in the imports of related seafood. In August 2023, China also completely suspended the import of seafood from Japan.

With the continuous economic growth and the improvement of residents’ living standards, Chinese consumers are increasingly demanding higher quality and safety for seafood. This has prompted the aquaculture industry to continuously innovate technologically and upgrade its industrial structure to meet market demands. The Chinese government attaches great importance to the quality and safety supervision of seafood, and has enacted a series of policies and regulations aimed at ensuring the highest standards of quality and safety of seafood to protect the rights and interests of consumers.

#### The threat posed by treated water to the seafood trade.

The Fukushima contaminated wastewater discharge incident in Japan has aroused widespread concern within the international community about the radioactive pollution of seafood, posing a potential threat to China’s seafood import trade. At the same time, the potential threat of nuclear radiation to seafood safety has affects the confidence of consumers in seafood, which could in-turn impact the import volume and price of seafood.

The potential threat of nuclear radiation to the safety of seafood cannot be ignored. It not only impairs the quality of seafood, but also directly endanger human health. The report by International Atomic Energy Agency (IAEA) indicates that the radioactive treated water contains radioactive substances such as cesium-137 and strontium-90. The International Commission on Radiological Protection (ICRP) has identified 100 mSv as a threshold level where there may be an increased risk of cancer. However, consuming even a low level of radioactive material still carries risks of cancer. Such as Cesium-137 potentially causing bone tissue sarcomas and leading to leukemia. Iodine-129 could easily lead thyroid cancer; and carbon-14 may damage human DNA [8].

If directly discharged inadequately treated radioactive wastewater into the sea without any sufficient treatment, it poses a potential threat to marine ecosystems, potentially causing genetic mutations, reproductive and developmental issues in marine life, and increasing the risk of cancer in organisms, including humans. Research indicates that radioactive isotopes from contaminated wastewater may accumulate through the marine food chain, leading to higher concentrations in seafood. This accumulation can impact the quality and safety of seafood, posing potential health risks to consumers.

Following the 2011 Fukushima nuclear disaster in Japan, seafood was found to have radioactive contamination levels exceeded international safety standards, leading to panic in the international market and the implementation of import restrictions on Japanese products by countries such as China.

In the backdrop of contaminated wastewater discharge, China’s import trade in seafood faces increased risks.

Moreover, consumers’ concerns about the safety of seafood may prompt them to choose alternatives, thereby affecting the overall demand of the seafood market. In the face of such challenges, in addition to strengthen the radiation detection of imported seafood to ensure food safety, actively exploring new sources of seafood supply to reduce dependence on a single market could be considered. The Chinese government has attached great importance to the use of scientific analytical models to evaluate and predict changes of seafood trade, to guarantee the sustainability of seafood imports and the dietary safety of the nation.

### Changes in China’s seafood import trade of Southeast Asia

#### The proportion of the trade volume of seafood imported from Japan.

China, as one of the world’s largest consumers of seafood, has kept a high level of import volume of the Japanese seafood before the discharge of contaminated wastewater. Utilizing the data from GACC, this paper has compiled trade data regarding China’s imported seafood for the year of 2022 and the data for China’s imported seafood after Japan’s treated water discharge (S3 Table), as shown in Table 1.

**Table 1:** China trade changes in imports of Japanese seafood.

Year	Total of seafood imported of China	Total of seafood imported from Japan to China	Ratio
Jan, 2022-Dec, 2022	$19.9 billion	$5.74 billion	2.88%
Sep, 2023-Aug, 2024	$18.2 billion	$0.0003billion	

As shown in Table 1, from September 2023 to August 2024, China imported Japanese seafood, with an actual trade volume amounting to $332,106, representing an insignificant percentage. It should be noted that China’s imports of Japanese seafood are mainly for ornamental fish series, such as freshwater fish, trout, eels, carp, Atlantic and Pacific bluefin tunas, etc.

According to Table 1, the total value of various seafood imported into China exceeded $19.9 billion in 2022, with $5.74 billion imported from Japan, accounting for 2.88% of China’s total seafood imports. By classifying and retrieving data from GACC, it is evident that shrimp, crab, and shellfish comprise 58.52% of the Japan’s seafood exports to China, representing the largest trade volume; frozen fish, fresh and cold fish follow, with a proportion of 36.29%.

On the same day that the Japanese government announced the full discharge of contaminated water from Fukushima into the ocean in July 2023, GACC made a decision to prevent contaminated Japanese food from flowing to China [5]. The Chinese customs decided to ban the import of seafood from 10 prefectures and cities in Japan impacted by radioactive treated water, while implementing 100% from other parts of Japan food inspection. They would strictly verify the relevant documentation and strengthen the detection of radioactive substances.

After the decision began, the total volume of seafood imported from Japan dropped sharply in July. Imports of fresh bluefin tuna dropped by 47% year-on-year, and the import of fresh scallops saw a dramatic decline of 98% compared to the same period last year. In addition to bluefin tuna and scallops, other seafood products such as abalone, eel, Arctic surf clam, fresh sea urchin, sea cucumber, oysters and other seafood products are subject to import restrictions [9].

With the implementation of Japan’s plan to discharge nuclear wastewater in August,China has suspended the import of Japan’s seafood. According to the latest import data from September 2023 to August 2024, China’s import volume of seafood were $18.2 billion, down 8.27 percent year-on-year. This change not only reflects the markets concerns about nuclear radiation pollution, but also shows a decline in consumer confidence in seafood.

#### Trade changes of seafood imported from Southeast Asia.

According to the official data from GACC, the top 10 countries in terms of China’s seafood import trade volume from 2018 to 2023 were Ecuador, Russia, Canada, India, Vietnam, the United States, Indonesia, Norway, New Zealand, Chile. Japan ranked 13^th^. Detail information can be founded in Table 2.

**Table 2:** The top 20 countries of China’s seafood import trade from 2018 to 2023.

Rank	country	Total amount	Rank	country	Total amount
1	Ecuador	12072871222	11	Thailand	2171722072
2	Russia	11438217286	12	Australia	2085157158
3	Canada	5530965191	13	Japan	2074502301
4	India	5237046893	14	Peru	1710560272
5	Vietnam	5236971416	15	Argentina	1430501618
6	United States	4919920882	16	Greenland	1426077873
7	Indonesia	4701236867	17	Malaysia	1360355047
8	Norway	3638272478	18	South Korea	1162424711
9	New Zealand	2327146720	19	Mexico	987438122
10	Chile	2187674711	20	Pakistan	857240924

Unit: US dollars .(Data source: GACC, http://stats.customs.gov.cn/).

According to the statistics, Southeast Asian countries had astonishing import trade volumes, such as Vietnam at 5.2 billion, Indonesia at 4.7 billion, Thailand at 2.1 billion and Malaysia at 1.3 billion US dollars.

Since Japan’s nuclear pollutant discharge, the seafood import trade is also quietly changing. On the one hand, China has intensified the scrutiny of radioactive substances in imported seafood, elevating the import standards and leading to the rejection of Japanese seafood for consumption. On the other hand, Chinese importers of seafood have begun to seek seafood from other countries as alternatives. In addition to the landlocked country of Laos, the trade volume of 10 countries, significant changes in trade volume have also occurred in the other 10 countries of Southeast Asia, including Vietnam, Cambodia, Thailand, Myanmar, Malaysia, Singapore, Indonesia, Brunei, the Philippines, and Timor-Leste. The trade changes of seafood exported from Southeast Asia to China are shown in Table 3.

**Table 3:** Changes of seafood trade volume imported from 10 Southeast Asian countries.

Country	Total amount (2018-2023)	Average (2018-2023)	Total amount (2023-2024)	Changes in trade volume
Vietnam	5236971416	1047394283	917557214	−129837069**↓**
Indonesia	4701236867	940247373	1185018164	244770791**↑**
Thailand	2171722072	434344414	424253123	−10091291**↓**
Malaysia	1360355047	272071009	275489385	3418376**↑**
Burma	638730800	127746160	192508781	64762621**↑**
Philippines	504791489	100958298	111380720	10422422**↑**
Singapore	88755891	17751178	26863926	9112748**↑**
Brunei	7168410	1433682	349967	−1083715**↓**
Timor-Leste	204750	40950	0	−40950**↓**
Cambodia	67853	13571	313113	299542**↑**
Total Southeast Asia	14710004595	2942000919	3133734393	191733474**↑**
Global total	81551413960	16310282792	18288313858	1978031066**↑**

Unit: US dollars. (Data source: GACC, http://stats.customs.gov.cn/).

According to Table 3, during the five years from 2018 to 2023, the average annual import seafood trade volume of China reached 16 billion, and the total trade volume of 10 Southeast Asian countries was about $2.9 billion, constituting 18% of the global total. Since August 2023, there has been an upward trend in the total volume of aquatic product exports from Southeast Asian countries to China, indicative of gradual increase in Southeast Asia’s market share. Notably China has increased the import of seafood from countries such as Indonesia, Myanmar, the Philippines.While there has been a significant decrease in imports from countries with larger market shares like Vietnam and Thailand.

#### Trade changes of seafood imported from other Asian countries.

The discharge of radioactive treated water has a profound impact on seafood export to China from Southeast Asian. However, there are 48 countries in Asia, and the trade from other Asian countries has mainly increased. Table 4 shows the trade changes of other Asian countries.

**Table 4:** Changes of seafood trade volume imported from other Asian countries.

Country/region		Average (2018-2023)	Total amount (2023-2024)	Changes in trade volume
East Asia	Taiwan, China	156164355	215605087	59440732**↑**
	Hongkong, China	149096001	269227340	120131339**↑**
	Mongolia	8965	46440	37475**↑**
	South Korea	232484942	281235486	48750544**↑**
South Asia	Bangladesh	53140689	86214653	33073964**↑**
	India	1047409379	1213960441	166551062**↑**
	Pakistan	171448185	204926249	33478064**↑**
	Sri Lanka	8637018	15039275	6402257**↑**
	Maldives	220103	0	−220103**↓**
Central Asia	Kazakhstan	2236874	3365673	1128799**↑**
West Asia	Iran	51514454	80641360	29126906**↑**
	Israel	5870	0	−5870**↓**
	Saudi Arabia	90478495	75233832	−15244663**↓**
	Oman	271012	0	−271012**↓**
	Yemen	19681	0	−19681**↓**
	Georgia	395	0	−395**↓**
	Turkey	13832977	10372972	−3460005 **↓**
Global total		16310282792	18288313858	1978031066**↑**

Unit: US dollars. (Data source: GACC, http://stats.customs.gov.cn/).

Among the 48 Asia countries, some did not export seafood to China, such as North Korea in East Asia; Bhutan and Nepal in South Asia; Kyrgyzstan, Tajikistan, Uzbek and Turkmen in Central Asia; and Afghanistan, Iraq, Syria, Jordan, Lebanon, Palestine, Bahrain, Qatar, Kuwait, Azerbaijan, Armenia, Cyprus, and the United Arab Emirates (U.A.E.) in West Asia. Table 4 lists all the countries that have had exported seafood to China since 2018, and it shows that some countries that previously exported seafood to China, such as Maldives, Israel, Oman, Yemen, and Georgia, did not export seafood to China in 2023–2024. These may have no trade exchanges or a reduction in trade exchanges, possibly due to the development of their own seafood industries, transportation distances, the political or economic cooperation between the two countries. Other countries or regions in East Asia, are almost all in a growth trend, such as South Korea, Mongolia, Bangladesh, India, Pakistan, Sri Lanka, Kazakhstan, and Iran.

#### Factors influencing Southeast Asian seafood export trade.

In the post-epidemic economic recovery period, there are many favorable factors for the export of Southeast Asian seafood to China.

Firstly, the positive growth of China’s economy has led to a recovery of seafood demand. China’s trade activities with the rest of the world have fully recovered to normal levels, providing a vast market for Southeast Asian seafood.

Secondly, Southeast Asia has a superior geographical location with lower logistics costs, which is highly appealing. Traditionally, seafood such as fish, shrimp and crabs from Southeast Asia have been favored by Chinese consumers. Additionally, China’s cessation of seafood imports from Japan has opened up new avenues for nations such as Indonesia, the Philippines. Moreover due to the Red Sea incident, shipping costs have increased, leading to a decline in China’s shrimp import from Ecuador and even markets in the Americas, Europe and other markets has decreased, whereas exports from Southeast Asia have increased.

However, the trade volume of aquatic product exports from individual Southeast Asian countries such as Vietnam and Thailand has shown negative growth. The primary reason for the emphasis on product quality in China’s food exports is the government’s stringent focus on food safety, which is a critical factor among all positive influences.

Since the implementation of Japan’s contaminated wastewater discharge plan, the Chinese government has suspended the import of seafood from Japan to protect the public health. However, in an attempt to alleviate the economic pressure caused by the reduction in Chinese imports of seafood, the Japanese government has planned to export nuclear-contaminated food to some countries such as Vietnam. The plan has not only aroused domestic controversy and discussion in Vietnam but also attracted the attention from the international community and Chinese consumers. In the face of the Japanese governments move that shifts domestic problems and endangers the food safety of the international community, Russia announced restrictions on the import of Japanese seafood, and the Chinese government is also paying high attention to the food safety of its citizens.

Secondly, the quality issues of seafood from Vietnam affect the market choices. Frozen seafood from Vietnam to China have been repeatedly rejected and returned for many times due to substandard quality inspections when exported to China. For example, in August 2019, Vietnam’s exports of seafood to China were seriously excessive polyphosphate, leading to 15 Vietnam’s seafood companies being blacklisted by China and banned from selling seafood to China.

Additionally some Vietnamese companies, despite the good reputation and sales, have indulged in misconduct targeting against Chinese exports, which has undermined the confidence of Chinese domestic consumers in products such as Vietnamese Basa fish. During the pandemic, Vietnamese seafood have been repeatedly rejected due to the detection of COVID-19 and other returned. In March 2022, the Vietnamese National Agro-Forestry-Fisheries Quality Assurance Department (Nafiqad) stated that as many as 52 batches of seafood exported from 36 Vietnamese enterprises to China were found to carry the SARS-CoV-2 virus and were consequently returned. The virus was detected in the packaging, containers and seafood. This behavior that damaged the image of Vietnamese seafood has impacted Vietnam’s export trade.

Food safety is a global issue that requires worldwide cooperation and coordination. The reduction in seafood exports from countries such as Vietnam is sometimes expected.

### Export strategies for Southeast Asian seafood to China

With the globalization deepening, China’s seafood import trade has achieved significant growth over the past few decades. However Japan’s radioactive treated water discharge event has cast an uncertainty shadow over this prosperous seafood trade scenario. Despite the Japanese government’s claim that the radioactive water has been treated in line with international standards, concerns from the international community persist, undoubtedly shaking consumers’ confidence in the safety of seafood.

**Fig 1:**
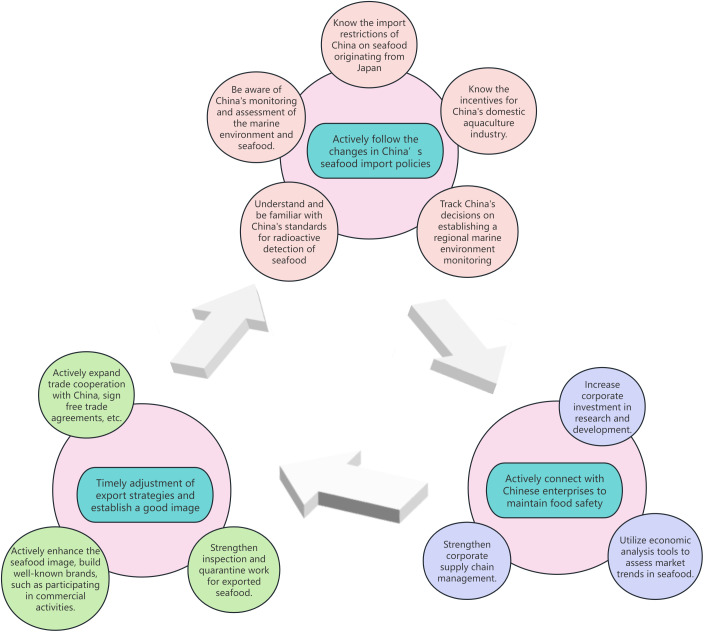
The flowchart of the proposed strategies.

As a major global consumer of seafood worldwide, China’s import demand will continue to be driven by the growth trend in domestic consumption and adjustments in import policies. Forecasting the future trend of China’s seafood import trade needs to consider multiple factors. Therefore, we have drawn up a flowchart of the proposed strategies (Fig 1) for better organization and clarity.

#### Actively follow the changes in China’s seafood import policies.

In response to the potential threat posed by Japan’s discharge of radioactive treated water to the safety of seafood, the Chinese government has implemented a series of strategies, to ensure public health and maintaining the stable development of seafood import trade.

Firstly, the Chinese government has strengthened radioactive detection for imported seafood to ensure that all imported products meet international safety standards. According to the data from General Administration of Quality Supervision, Inspection and Quarantine of the People’s Republic of China (AQSIQ), since the radioactive treated water discharge incident, the frequency of radioactive testing for Chinese seafood exported to Japan or subsequently re-exported to China has been increased by 100% to ensure the safety of Japanese food exported to China and o strictly prevent radioactive contaminated Japanese food from entering the Chinese market [10].

Secondly, the Chinese government communicated with the Japanese government through diplomatic channels, requesting it to furnish detailed data regarding radioactive treated water discharge, along with monitoring reports, to assess the long-term impact on the marine environment and seafood safety. The Chinese side has conducted over 10 rounds of intensive negotiations and consultations with Japan and relevant international organizations. On September 20 in 2024 year, China successfully promoted Japan to accept the long-term international monitoring of nuclear-contaminated water and with independent sampling by China [11]. China actively participates in relevant activities of the International Atomic Energy Agency (IAEA) to ensure that the supervision and assessment of the treated water discharge by the international community are transparent and scientific.

At the policy level, the Chinese government has adjusted the import trade policy of seafood and implemented stricter import restrictions on seafood from Japan. According to GACC, importation of Japanese seafood to China have decreased by about 30% since the radioactive treated water discharge incident, which not only reflects the governments emphasis on the health of its citizens but also demonstrates respect for international law and international obligations.

Furthermore, the Chinese government also encourages the development of the domestic seafood breeding industry to reduce its dependence on imported seafood. China promotes financial subsidies and technical support to promote the modernization and scale of domestic seafood aquaculture, which has improved the quality and output of domestic seafood. This strategy not only helps to ensure the supply security of domestic seafood but also lays the foundation for the long-term and stable development of seafood import trade.

Finally, by strengthening international cooperation and information exchange, the Chinese government, in partnership with with neighboring countries, jointly addresses potential marine environmental issues arising from the discharge of radioactive treated water. By establishing a regional marine environment monitoring network, China and its neighbors jointly monitor the levels of marine radioactivity to ensure timely detection and response to potential environmental risks. This strategy, based on cooperation and mutual benefit, not only helps to protect the Marine environment but also promotes political trust and economic cooperation within the region.

Facing the challenges brought by radioactive treated water discharge, the Chinese government has implemented a rang of countermeasures, such as strengthening communication with Japan and taking effective measures to safeguard the seafood safety. At the same time, it has increased the support for the domestic seafood industry, and encouraged the development of the domestic seafood breeding industry to reduce the dependence on imported seafood.

In summary, Southeast Asian seafood exports actively monitor the changes in China’s seafood import policies, particularly the radioactive detection standards such as GB/T11713-2015 [12] and GB/T16145-2022 [13], to ensure their products meet the stringent safety requirements set by the Chinese government. And they should actively pay attention to import restrictions on Chinese seafood and the development of China’s domestic aquaculture industry to maintain compliance with Chinese and international safety standards.

#### Timely adjustment of export strategies and establish a good image.

The discharge of contaminated wastewater by Japan has triggered a series of butterfly effects. According to the Table 2 of the seafood trade data shown above, following China’s suspension of the Japan market, it is evident that the seafood export trade from Southeast Asian countries such as Indonesia, Myanmar and the Philippines and other Southeast Asian countries to China increased significantly. Facing the adjustments of China’s seafood import trade strategies, Southeast Asian countries should timely adjust their export policies to accommodate the continually evolving market demands with high safety and quality seafood exports.

Firstly Southeast Asian countries should actively expand trade cooperation in seafood with China, and establish a closer cooperation mechanism by signing free trade agreements and strengthening bilateral trade relations. This not only provides a vast market space and opportunities for the countries in Southeast Asia, but also offers China a more diversified source of seafood.

Secondly, it should actively enhance the image of Southeast Asian seafood and enhance the Chinese consumers’ awareness and acceptance. By actively participating in various expos in China, or hosting seafood promotion events and food festivals, we can introduce the characteristics and advantages of Southeast Asian seafood to a wide range of consumers, including those in China, thereby promoting the sales of Southeast Asian seafood in the Chinese market. At the same time, governments should actively encourage domestic enterprises to establish long-term cooperative relationships with Chinese seafood suppliers to ensure the stability of the supply chain and product diversity.

Furthermore at the regulatory level, governments of various countries ought to stay updated on China’s seafood import policies and strengthen the inspection and quarantine work for imported seafood, and guarantee that all imported seafood meet China’s food safety standards. By improving the detection technologies and strengthening the regulatory efforts, the potential food safety risks in bilateral trade can be effectively prevented, the health rights and interests of consumers can be guaranteed, and the credibility and stability of the market can be maintained.

Despite facing numerous challenges, with effective policy guidance and flexible market adjustments, Southeast Asia’s aquatic product export trade is expected to maintain its growth momentum, while placing greater emphasis on improving product quality and ensuring consumer safety.

Therefore, to expand the market in China, Southeast Asian countries must promptly adjust their export strategies and actively enhance the image of seafood. This can be achieved by participating in trade fairs and promotional activities to raise awareness among Chinese consumers about Southeast Asian seafood. Furthermore, Southeast Asian governments can encourage enterprises to establish long-lasting cooperative partnerships with Chinese suppliers. With policy guidance and market adjustments, the export of Southeast Asian seafood is expected to continue growing.

#### Actively connect with Chinese enterprises to maintain food safety.

In the face of the potential negative impacts on seafood trade due to Japan’s discharge, it is crucial for corporations in China and Southeast Asia to implement measures to mitigate these impacts.

First of all, enterprises must strengthen supply chain management to ensure the safety and reliability of seafood. Enterprises need to strengthen management and strictly control the sources of seafood. This includes proactively creating a traceability system for seafood to ensure that each batch of seafood can be traced to their original fishing locations, preventing contaminated seafood from entering the Chinese market, and maintaining market stability as well as the health and safety of consumers.

Secondly, enterprises should boost their investments in research and development to develop advanced detection technologies.It will enhance the precision of the detecting radioactive substances in seafood and guarantee that the products meet international safety standards. Strengthen collaborations with scientific research institutions to jointly develop novel anti-fouling technologies to mitigate the impact of marine pollution on seafood safety. Through close cooperation with professional institutions, enterprises can stay informed about the latest scientific research results and detection methods, so as to control the pollution risks at the source and ensuring the quality and safety of seafood.

Furthermore, enterprises can utilize economic analysis tools such as gravity models to evaluate the attractiveness of different markets to seafood, adjust their market strategies accordingly, explore new markets, and reduce the dependence on a single market. In terms of marketing strategies, enterprises should reinforce brand building and bolster consumers’ confidence of seafood. Collaborate with third-party certification bodies to obtain product security certification to enhance consumer trust. Launch the “Safe Seafood” label to transparently demonstrate the safety of products to consumers.

In conclusion, enterprises in China and Southeast Asian countries should adopt a comprehensive approach to the radioactive treated water discharge, focusing not only on avoiding short-term risks but also on the long-term market development and brand building. We should strengthen international cooperation and improve regulatory levels, while taking into account the stable development of seafood import trade. These measures help to address current environmental threats and lay a solid foundation for long-term cooperation between China and Southeast Asian in the trade of seafood for the future. Looking forward, China’s seafood import trade will give greater emphasis to security and sustainability. At the same time, the international community will make joint efforts to ensure the safety of the marine environment and ensure a healthy supply of global seafood.

## Conclusion

After the Fukushima nuclear accident in Japan, the issue of contaminated wastewater discharge has been a focus point of the international community, especially it has a profound impact on China’s import trade of seafood. The discharge contaminated wastewater may potentially cause a long-term negative impact on the Marine ecosystem, and affect the safety and quality of seafood through the food chain.The import volume of related seafood in China has significantly declined, reflecting consumer concerns about seafood safety and the market response to potential risks. Facing challenges brought by Japan’s radioactive treated water discharge affecting China’s import and Southeast Asia’s exports, emphasis should be placed on strengthening international cooperation, improving testing standards, optimizing supply chain management, and promoting the upgrading of the domestic seafood industry. Southeast Asian trading partners should pay close attention to the changes in these factors and flexibly adjust their strategies to cope with potential market fluctuations in the future.

## Supporting information

S1 Table Trade volume of the world exported seafood to China from 2018 to 2023. The annual average trade volume of seafood exported to China is shown in Table 1 from 115 countries over the five years from September 2018 to August 2023. The table is arranged in descending order of trade volume. Among these, we have marked the ten countries of Southeast Asia, namely Vietnam, Indonesia, Thailand, Malaysia, Myanmar, Philippines, Singapore, Brunei, Timor-Leste, and Cambodia, in red for the convenience of readers to view.
(XLSX)

S2 Table Trade volume of the world exported seafood to China from 2023 to 2024. The trade volume of seafood exported is shown from 88 countries to China from September 2023 to August 2024, following the discharge of nuclear wastewater from Japan. The table is arranged in descending order of trade volume. Among these, we have marked the nine countries in Southeast Asia, namely Indonesia, Vietnam, Thailand, Malaysia, Myanmar, Philippines, Singapore, Brunei, and Cambodia, in red for the convenience of readers.
(XLSX)

S3 Table Trade volume of Japan exported seafood to China in 2022. The Table 3 presents the trade situation of Japanese seafood exported to China in 2022. It details the export categories and trade volumes of various seafood and their by-products to China.
(XLSX)
