# Correction: Analysis of the development trend of Chinese seafood imports from Southeast Asia after the Fukushima-Daiichi radioactive treated water discharge from Japan

**DOI:** 10.1371/journal.pone.0340127

**Published:** 2026-01-02

**Authors:** Xiaojun Qi, Liguo Gao

The authors, Xiaojun Qi and Liguo Gao, should be noted as shared co-corresponding authors on this work. Liguo Gao’s email address is: 429026719@qq.com.
